# Age-dependent sex differences in calcium and phosphate homeostasis

**DOI:** 10.1530/EC-20-0509

**Published:** 2021-02-04

**Authors:** W N H Koek, N Campos-Obando, B C J van der Eerden, Y B de Rijke, M A Ikram, A G Uitterlinden, J P T M van Leeuwen, M C Zillikens

**Affiliations:** 1 Department of Internal Medicine, Erasmus MC, University Medical Center, Rotterdam, the Netherlands; 2 Department of Clinical Chemistry, Erasmus MC, University Medical Center, Rotterdam, the Netherlands; 3 Department of Epidemiology, Erasmus MC, Rotterdam, the Netherlands

**Keywords:** calcium, phosphate, ageing, sex differences

## Abstract

**Background:**

Sex differences in calcium and phosphate have been observed. We aimed to assess a relation with age.

**Methods:**

We used the laboratory values of serum calcium, phosphate and albumin from three different samples ( 2005, 2010 and 2014 years) using the hospital information system of Erasmus MC, Rotterdam. The samples were divided into three age groups: 1–17, 18–44 and ≥45 years. Sex differences in calcium and phosphate were analyzed using ANCOVA, adjusting for age and serum albumin. Furthermore, sex by age interactions were determined and we analyzed differences between age groups stratified by sex.

**Results:**

In all three samples there was a significant sex × age interaction for serum calcium and phosphate, whose levels were significantly higher in women compared to men above 45 years. No sex differences in the younger age groups were found. In men, serum calcium and phosphate levels were highest in the youngest age group compared to age groups of 18–44 and ≥45 years. In women, serum calcium levels were significantly higher in the age group 1–17 and the age group ≥45 years compared to the 18–44 years age group. In women, serum phosphate was different between the three different age groups with highest level in the group 1–17 years and lowest in the group 18–44 years.

**Conclusion:**

There are age- dependent sex differences in serum calcium and phosphate. Furthermore, we found differences in serum calcium and phosphate between different age groups. Underlying mechanisms for these age- and sex- differences are not yet fully elucidated.

## Introduction

Calcium and phosphate imbalance has been linked to several disorders such as cardiovascular disease, metabolic syndrome, osteoporosis and mortality (1, 2, 3, 4). Some of these conditions display marked sex-specific incidences (3, 5, 6), which might be related to sex differences in calcium and phosphate homeostasis. In a population-based study (W N H Koek *et al.*, unpublished observations) we found postmenopausal women to have higher serum calcium and phosphate levels compared to men above 45 years of age. Adjustment for serum testosterone levels diminished the sex differences in serum phosphate but not serum calcium levels, while adjustment for serum estradiol did not consistently influence sex differences in serum calcium and phosphate.

In this study we aimed to evaluate whether the observed sex differences in men and women ≥45 years of age exist at younger age, and how serum levels differ between different age groups.

We, therefore, compared serum calcium and phosphate levels and calcium × phosphate in three age groups of men and women, using three different samples derived from the Hospital Information System (HIS) of the Erasmus MC, with age ranging from 1–97 years.

## Materials and methods

The data from the study population were derived from the HIS of Erasmus MC. We sampled three different years 2005, 2010 and 2014. Subjects were included that visited the hospital either in out-patient setting or while being admitted, and in whom serum calcium, phosphate and albumin levels were measured. Per subject only the first serum values measured were included in the analyses. The laboratory values were not coupled to diagnosis, reason for admission or out-patient clinic visit. Only the first serum measurements where serum calcium, serum phosphate and serum albumin were determined at the same time were selected for analyses. The groups were divided into three different age categories: a group aged 1–17 years old, representing youth; a group aged 18–44 years old, representing adolescence and young adulthood; a group above ≥45 years. The latter group is based on our previous study where we addressed sex differences in calcium homeostasis (W N H Koek *et al.*, unpublished observations) which was done in a population-based cohort study where subjects were included that were 45 years or older (7). Furthermore, 45 years is the age when hormonal changes associated with natural menopause first appear (8, 9). The total number of subjects in the different groups are depicted in Table 1 and included 4074 subjects in 2005, 4708 subjects in 2010 and 6433 subjects in 2014. In the 2014 sample, four subjects had assigned dates of birth on January 1,1900 since their real date of birth was unknown; therefore, these subjects were excluded from the analyses. Since the data were anonymized and there was the possibility of including subjects three times when all samples would be merged, we chose to analyze the three samples separately. The medical ethical committee of the Erasmus MC approved the study.

**Table 1:** Calcium, phosphate and albumin levels in men and women in three different age groups in 2005, 2010 and 2014.

	1–17 years			18–44 years			≥45 years		
	Men	Women	*P*-AN(C)OVA	Men	Women	*P*-AN(C)OVA	Men	Women	*P*-AN(C)OVA
2005									
Number	249	225		540	510		1446	1104	
Age (years)	9.29 (8.64–9.94)	9.67 (9.04–10.29)	0.42	32.85 (32.19–33.52)	33.20 (32.50–33.90)	0.48	61.66 (61.17–62.16)	61.89 (61.27–62.52)	0.57
Albumin (g/L)	43.02 (42.24–43.79)	42.65 (41.70–43.60)	0.24	42.40 (41.87–42.94)	41.43 (40.92–41.93)	0.009	40.55 (40.23–40.87)	40.70 (40.35–41.06)	0.48
Calcium (mmol/L)	2.409 (2.382–2.437)	2.398 (2.373–2.424)	0.98	2.348 (2.330–2.365)	2.336 (2.320–2.351)	0.36	2.321 (2.312–2.331)	2.352 (2.341–2.363)	3.6 × 10^-7^
Calcium corrected (mmol/L)	2.389 (2.369–2.410)	2.385 (2.369–2.402)	0.87	2.339 (2.326–2.353)	2.347 (2.336–2.359)	0.38	2.350 (2.344–2.357)	2.378 (2.370–2.387)	3.6 × 10^-7^
Phosphate (mmol/L)	1.554 (1.515–1.592)	1.546 (1.494–1.598)	0.96	1.115 (1.087–1.144)	1.111 (1.084–1.138)	0.77	1.093 (1.076–1.110)	1.167 (1.151–1.183)	1.7 × 10^-9^
Calcium× phosphate (mmol^2^/L^2^)	3.750 (3.647–3.853)	3.721 (3.593–3.850)	0.87	2.613 (2.547–2.679)	2.589 (2.527–2.650)	0.91	2.528 (2.490–2.567)	2.747 (2.708–2.787)	2.1 × 10^-14^
2010									
Number	157	119		591	580		1887	1374	
Age (years)	9.36 (8.54–10.17)	10.67 (9.66–11.68)	0.04	33.64 (32.98–34.30)	33.37 (32.73–34.01)	0.54	63.06 (62.57–63.49)	63.48 (62.90–64.07)	0.23
Albumin (g/L)	41.94 (40.78–43.09)	41.78 (40.54–43.02)	0.76	42.73 (42.10–43.35)	41.70 (41.15–42.25)	0.013	40.22 (39.89–40.56)	40.82 (40.46–41.19)	0.012
Calcium (mmol/L)	2.337 (2.307–2.367)	2.332 (2.302–2.362)	0.60	2.288 (2.273–2.303)	2.259 (2.244–2.273)	0.19	2.247 (2.236–2.257)	2.288 (2.277–2.298)	2.0 × 10^-6^
Calcium corrected (mmol/L)	2.336 (2.286–2.386)	2.328 (2.279–2.377)	0.95	2.303 (2.276–2.329)	2.253 (2.229–2.277)	0.005	2.211 (2.196–2.227)	2.264 (2.248–2.280)	3.0 × 10^-6^
Phosphate (mmol/L)	1.434 (1.387–1.481)	1.393 (1.308–1.479)	0.91	1.071 (1.044–1.098)	1.086 (1.064–1.109)	0.61	1.069 (1.053–1.085)	1.136 (1.121–1.152)	1.4 × 10^-9^
Calcium × phosphate (mmol^2^/L^2^)	3.362 (3.240–3.485)	3.258 (3.069–3.456)	0.99	2.437 (2.378–2.496)	2.451 (2.400–2.503)	0.57	2.388 (2.354–2.421)	2.598 (2.563–2.634)	7.6 × 10^-16^
2014									
Number	296	254		708	687		2543	1941	
Age (years)	9.61 (9.01–10.21)	10.66 (10.03–11.29)	0.013	33.40 (32.82–33.98)	32.98 (32.40–33.57)	0.33	63.74 (63.36–64.13)	63.22 (62.76–63.69)	0.093
Albumin (g/L)	40.51 (39.65–41.37)	41.05 (40.10–41.99)	0.57	42.64 (42.11–43.16)	40.91 (40.44–41.39)	2.0 × 10^-6^	38.95 (38.67–39.23)	39.92 (39.61–40.23)	2.1 × 10^-5^
Calcium (mmol/L)	2.338 (2.316–2.359)	2.347 (2.322–2.373)	0.60	2.345 (2.331–2.355)	2.300 (2.287–2.341)	0.13	2.283 (2.275–2.291)	2.334 (2.325–2.343)	1.0 × 10^-12^
Calcium corrected (mmol/L)	2.368 (2.353–2.382)	2.366 (2.347–2.386)	0.64	2.332 (2.323–2.342)	2.322 (2.313–2.332)	0.11	2.344 (2.338–2.350)	2.376 (2.369–2.382)	5.7 × 10^-13^
Phosphate (mmol/L)	1.412 (1.377–1.446)	1.366 (1.323–1.409)	0.33	1.051 (1.027–1.076)	1.043 (1.022–1.064)	0.16	1.044 (1.031–1.057)	1.115 (1.102–1.128)	3.2 × 10^-17^
Calcium × phosphate (mmol^2^/L^2^)	3.310 (3.224–3.396)	3.203 (3.104–3.301)	0.28	2.453 (2.340–2.506)	2.399 (2.350–2.499)	0.20	2.369 (2.341–2.396)	2.594 (2.565–2.623)	8.3 × 10^-27^

Values are depicted as means with 95% confidence intervals between brackets.

### Assay methods

Serum samples from subjects were analyzed at the department of clinical chemistry of Erasmus MC Rotterdam, the Netherlands.

Serum albumin, calcium and inorganic phosphate were measured using the Hitachi 917 Analyzer in 2005, the Modular system in 2010 and Cobas 8000 in 2014 (Roche). Calcium × phosphate (mmol^2^/L^2^) was a product of the multiplying the subjects’ serum calcium and phosphate level. Serum calcium corrected for albumin levels was calculated using the formula: calcium (corrected) = calcium measured + 0.02 × (42 - albumin measured) (10).

### Statistical analyses

In order to minimize inferences of change in assay performance on our data sample years 2005, 2010 and 2014 were analyzed separately. Sex differences between serum calcium and phosphate were tested in the three different age categories as well as in decades using analysis of covariance (ANCOVA) within a general linear model with either serum calcium, serum phosphate or calcium × phosphate as dependent variables and sex as a fixed factor. Total serum calcium includes calcium bound to albumin, calcium bound to other proteins and ionized calcium. Moreover, serum albumin levels are lower in critical ill patients and are correlated with disease status. Therefore, data were adjusted for age and serum albumin levels. As a sensitivity analysis, we used the formula as described previously in the assay methods, to correct individual serum calcium for their individual albumin levels. Furthermore, to exclude an impact of very high or low albumin levels on serum calcium levels, we performed sensitivity analyses restricting our data only to those subjects with albumin levels within a narrow range (38–42 g/L).

Sex differences in serum calcium corrected for albumin were assessed using ANCOVA within a general linear model with serum calcium corrected for albumin as described in assay methods as dependent variable with sex as a fixed factor. Since albumin was part of the formula, we adjusted the corrected serum calcium analyses only for age.

Differences between the age groups in serum calcium levels, corrected serum calcium levels, serum phosphate levels and calcium × phosphate were assessed stratified for by sex using ANCOVA in a general linear model with serum calcium, corrected serum calcium, phosphate and calcium × phosphate as dependent variables and age groups as a fixed factor. Analyses were adjusted for albumin, apart from the analysis for corrected serum calcium. The interaction between sex and age on serum calcium and phosphate was determined using linear regression analyses. Statistical analyses were performed using SPSS (version 23). Statistical significance was defined as a *P* < 0.05.

Potential non-linearity of the associations was tested through the comparison of linear models and natural cubic spline models. We used the STATA package uvrs –univariate regression spline– that selects the spline model for the covariate of interest that provides the best prediction for the outcome (11). When other covariates are added, they are adjusted linearly. By default, the spline regression applies three spline knots (m = 3) which are located in equally spaced centiles of the distribution of the covariate. We ran likelihood ratio tests (LRTs) to compare between models, as linear models (m = 0) are nested within spline models. Additionally, models were compared through Akaike information criteria (AIC) (12): the lower, the better fit.

Stata (version 15, College Station TX: Stata Corp LP) was used for spline regression models and plots.

## Results

### Sample characteristics

For the study, three samples from the HIS were collected. The 2005 sample contained 2235 men with an average age of 48–87 years and 1839 women with an average age of 47–55 years. The 2010 sample contained 2635 men with an average age of 53–25 years and 2073 women with an average age of 52–03 years. The 2014 sample contained 3547 men with an average age of 53–17 years and 2882 women with an average age of 51–38 years.

### Sex differences

**Figure 1:**
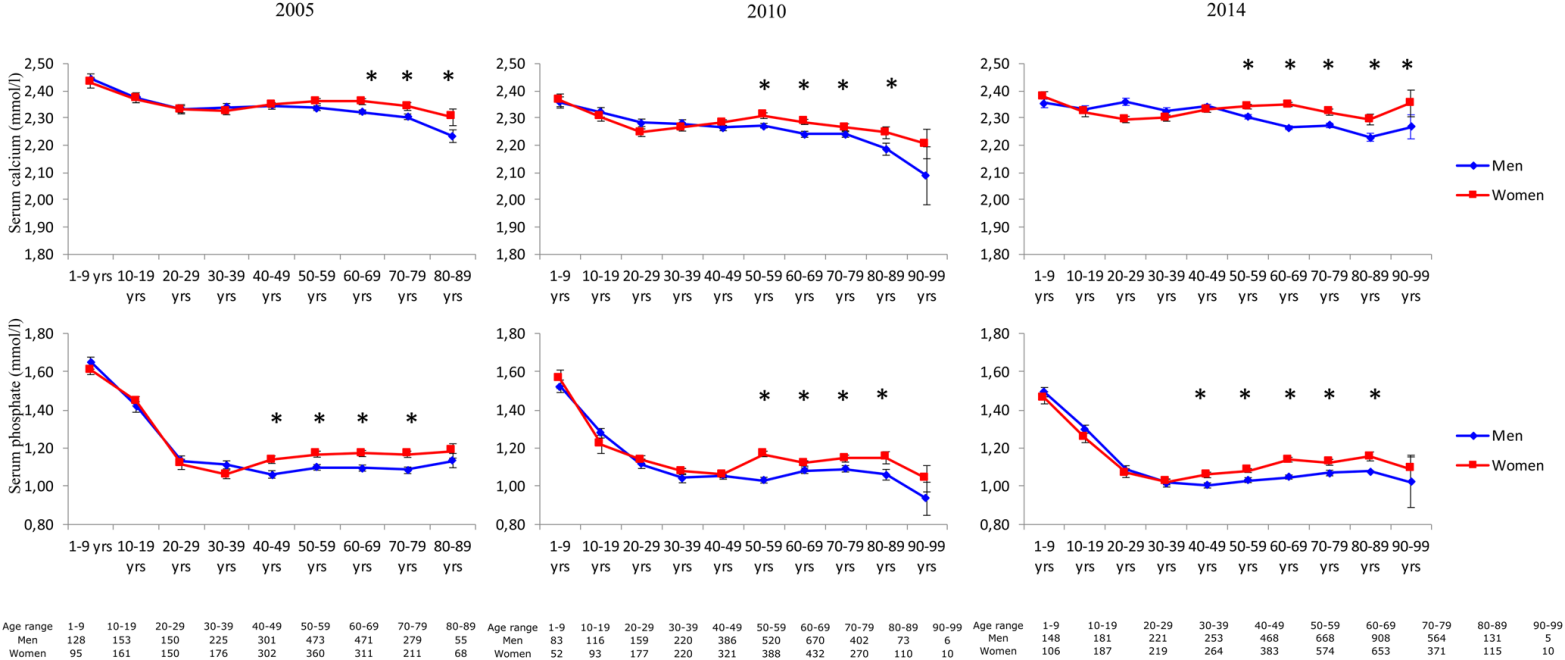
Line graphs of serum calcium and serum phosphate levels in men and women stratified per decade. *Depicts a significant sex difference. Furthermore, below figures is a table with the numbers per sex and per group.

In subjects of 45 years and older, women had higher serum calcium levels compared to men in all three samples (Table 1). As for serum calcium, women ≥45 years of age displayed higher serum phosphate levels compared to men (Table 1). As a consequence, calcium × phosphate was also higher. The age groups 1–17 years and 18–44 years of age showed no sex difference in serum calcium and phosphate levels in any of the three samples (Table 1). In Fig. 1 serum calcium and phosphate levels are shown per decade in men and women in the three years sampled. In the sample from 2005, there were only 10 men and women in the age group above 90, therefore these were not depicted in the figure. In the 2005 and in the 2014 samples, a significant sex difference in serum phosphate appeared in the 5th decade, whereas in the 2010 sample this appeared in the 6th decade. In the 2005 sample a significant sex differences appeared for serum calcium levels in the 7th decade whereas in the 2010 and 2014 sample this difference appeared in the 6th decade.

### Sensitivity analyses related to serum albumin levels

Sex differences in albumin levels were present in the age group 18–44 years in all three samples with men having higher albumin levels compared to women. However, in the 2010 and in the 2014 samples, men ≥45 years had lower serum albumin levels compared to women of the same age. In the youngest age groups in all samples no sex differences were found for serum albumin levels.

After correcting serum calcium for serum albumin levels according to the formula described in the material and methods, sex differences in serum calcium levels remained with women having higher corrected serum calcium levels compared to men in the age groups above 45 years of age (Data not shown).

Also, after restricting our analyses to subjects with albumin levels within the normal range (38–42 g/L), we found similar results (data not shown).

### Age differences in serum calcium and phosphate stratified by sex

**Figure 2:**
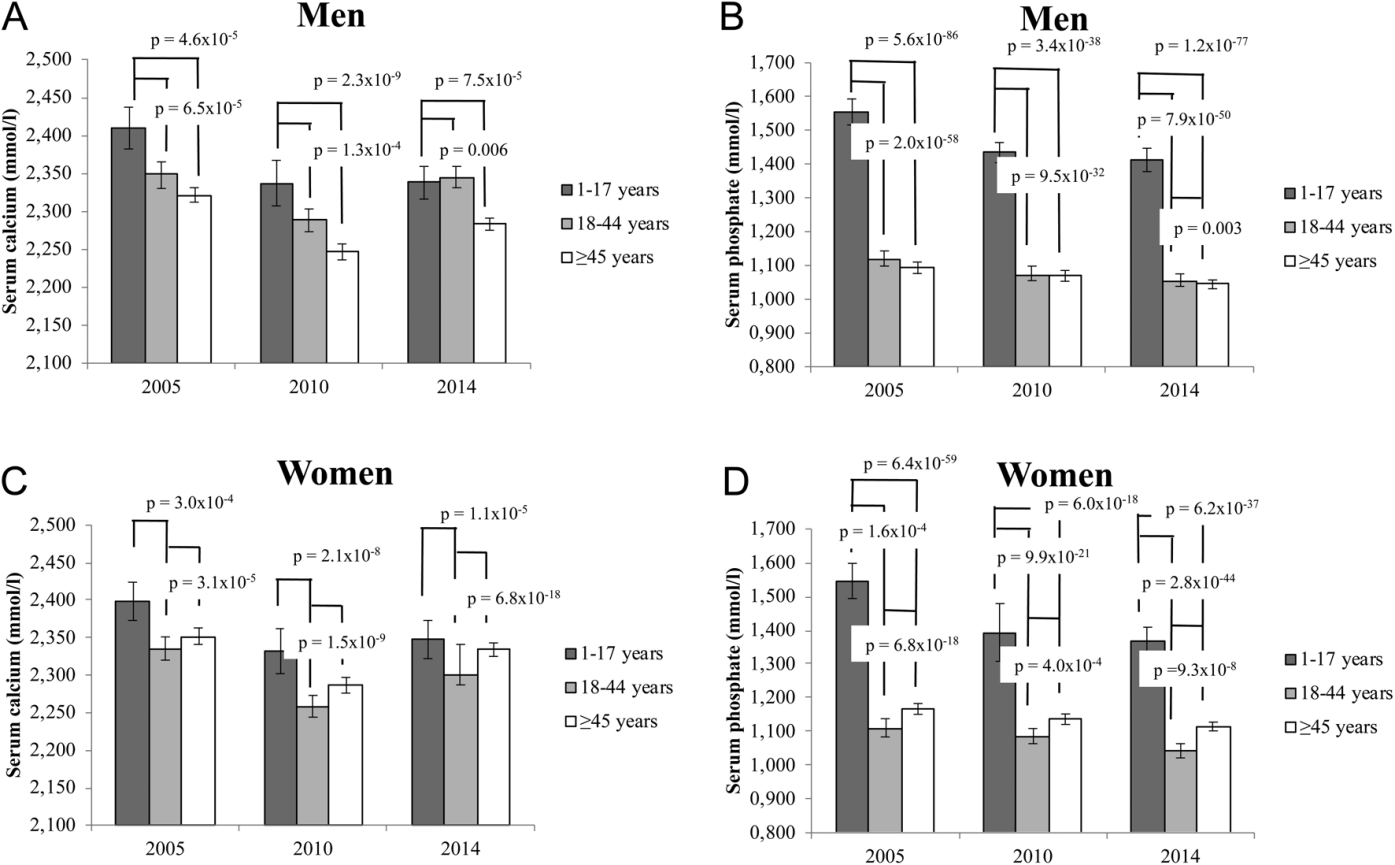
Serum calcium and phosphate levels in men and women stratified per age group. The T-bars denote standard errors.

Figure 2 depicts serum calcium and phosphate levels per age group stratified according to sex.

#### Men

In all 3 years samples men between 1 and 17 years of age had higher serum calcium compared to the age group ≥45 years (*P* = 4.6 × 10^−5^ in 2005, *P* = 1.3 × 10^−4^ in 2010 and *P* = 0.006 in 2014) and compared to the age group 18–44 years in 2005 (*P* = 6.5 × 10^−5^) and 2010 (*P* = 2.3 × 10^−9^) (Fig. 2A).

Serum phosphate levels were higher in the youngest age group compared to the age group 18–44 (*P* = 2.0 × 10^−58^, *P* = 9.5 × 10^−32^ and *P* = 7.9 × 10^−50^ for 2005, 2010 and 2014, respectively) and the group men ≥45 years of age (*P* = 5.6 × 10^−86^, *P* = 3.4 × 10^−38^ and *P* = 1.2 × 10^−77^ for 2005, 2010 and 2014, respectively, (Fig. 2B). Only in 2014 but not in 2005 or 2010 the group ≥45 years men had significantly higher serum phosphate levels compared to the adult group of 18–44 years (*P* = 0.003) (Fig. 2B).

#### Women

In 2005, 2010 and 2014 women in the age group 1–17 years had significantly higher serum adjusted calcium levels compared to 18–44 years and (*P* = 3.0×10^−4^, *P* = 2.1×10^−8^, and *P* = 1.1×10^−5^ for 2005, 2010 and 2014, respectively) (Fig. 2C). Women ≥45 years had significantly higher serum calcium levels compared to women in the age group 18–44 years (*P* = 3.1×10^−5^, *P* = 1.5×10^−9^ and *P* = 6.8×10^−18^ for 2005, 2010 and 2014, respectively) (Fig. 2C). There was no significant difference in serum calcium levels in women aged 1–17 compared to women ≥45 years (Fig. 2C).

Serum phosphate differed significantly between the three different age groups in all three samples. The age group 1–17 years had the highest serum phosphate levels whereas the age group 18–44 had lowest serum phosphate levels (Fig. 2D).

### Non-linearity

We found strong statistical evidence of non-linearity in the associations between calcium and phosphate levels and age across sexes and samples with only one exception –calcium levels in men of the 2010 sample. Results from LRTs and AIC are displayed in Table 2. Sex-stratified spline plots are displayed in Supplementary Fig. 1A, B and C (see section on supplementary materials given at the end of this article).

**Table 2:** Results of comparison between linear models and spline models.

	LRT	AIC linear model	AIC spline model
Sample: 2005			
Ca levels in men	0.002	*−1122*	*−1131*
Ca levels in women	<0.001	*−902*	*−920*
P levels in men	<0.001	1559	1356
P levels in women	<0.001	1017	788
Sample: 2010			
Ca levels in men	0.175	*−443*	*−443*
Ca levels in women	<0.001	*−895*	*−926*
P levels in men	<0.001	2120	1940
P levels in women	<0.001	1037	857
Sample: 2014			
Ca levels in men	0.012	*−1304*	*−1309*
Ca levels in women	<0.001	*−1056*	*−1082*
P levels in men	<0.001	2508	2165
P levels in women	<0.001	1633	1217

Italic font: note the negative values: the lower, the better fit.

AIC, Akaike information criteria; LRT, likelihood ratio test.

### Interaction between sex and age on sex differences in serum calcium and phosphate

In Table 3 interaction analyses of age and sex are shown. In all three samples there was a significant association of the sex × age interaction term with serum calcium, corrected serum calcium, serum phosphate and calcium × phosphate. In the three samples when addressing the sex × age interaction term, age or sex independently, were not consistently associated with serum calcium and phosphate levels.

**Table 3:** Summary of linear regression analyses with in the model selection for serum calcium, serum phosphate, calcium × phosphate and corrected serum calcium levels.

Parameters	Variables in the model	Regression coefficient (95% CI)	Standardized beta	*P* -value	R^2^ of the final model
2005					
Serum calcium (mmol/L)	Sex	−−0.0148 (−0.036/0.0069)	−0.039	0.18	
	Age	−0.000404 (−0.00069/−0.00012)	−0.044	0.005	
	Albumin	0.0204 (0.0197/0.0211)	0.671	0.0 × 10^0^	0.457
	Sex × age interaction	0.000686 (0.000273/0.00110)	0.100	0.001	
Serum phosphate (mmol/L)	Sex	−0.044789 (−0.096496/0.006919)	−0.064	0.09	
	Age	−0.005297 (−0.00597/−0.00462)	−0.318	1.2 × 10^−51^	0.2078
	Albumin	−0.00112 (−0.00278/0.00054)	−0.020	0.19	
	Sex × age interaction	0.00181 (0.00082/0.00279)	0.144	3.3 × 10^−4^	
Calcium × phosphate (mmol^2^/L^2^)	Sex	−0.12066 (−0.24159/0.00027)	−0.072	0.05	
	Age	−0.01332 (−0.01490/−0.01174)	−0.334	3.6 × 10^−59^	0.123
	Albumin	0.02074 (0.01686/0.02462)	0.156	2.4 × 10^−25^	
	Sex × age interaction	0.00512 (0.00282/0.00742)	0.170	1.4 × 10^−5^	
Corrected serum calcium (mmol/L)	Sex	−0.01510 (−0.03680/−0.006595)	−0.054	0.17	
	Age	−0.00043 (−0.00071/−0.000144)	−0.063	0.0031	0.007
	Sex × age interaction	0.00069 (0.00028/0.00111)	0.136	0.0011	
2010					
Serum calcium (mmol/L)	Sex	−0.02823 (−0.05511/−0.00134)	−0.066	0.04	
	Age	−0.00036 (−0.00069/−0.00004)	−0.033	0.03	0.423
	Albumin	0.01882 (0.01818/0.01946)	0.647	0.0 × 10^0^	
	Sex × age interaction	0.000843 (0.00036/0.00132)	0.115	5.8 × 1^−4^	
Serum phosphate (mmol/L)	Sex	−0.04493 (−0.10047/0.01061)	−0.066	0.113	
	Age	−0.003133 (−0.00381/−0.00246)	−0.178	1.4 × 10^−19^	
	Albumin	−0.00583 (−0.00714/−0.00451)	−0.126	5.3 × 10^−18^	0.036
	Sex × age interaction	0.001699 (0.00071/0.00269)	0.081	7.9 × 10^−4^	
Calcium × phosphate (mmol^2^/L^2^)	Sex	−0.13055 (−0.25391/−0.00718)	−0.086	0.04	
	Age	−0.00781(−0.00931/−0.00631)	−0.199	3.7 × 10^−24^	0.043
	Albumin	0.00865 (0.00572/0.01157)	0.084	7.2 × 10^−9^	
	Sex × age interaction	0.00494 (0.00274/0.00715)	0.190	1.1 × 10^−5^	
Corrected serum calcium (mmol/L)	Sex	−0.05961 (−0.11353/−0.00570)	−0.091	0.03	
	Age	−0.00271 (−0.00336/-0.00206)	−0.160	4.8 × 10^−16^	0.017
	Sex × age interaction	0.00156 (0.00060/0.00253)	0.139	0.002	
2014					
Serum calcium (mmol/L)	Sex	−0.03429 (−0.05360/−0.01497)	−0.085	5.0 x 10^−4^	
	Age	−0.000223 (−0.00046/0.00001)	−0.022	0.06	0.508
	Albumin	0.02000 (0.01951/0.02049)	0.709	0.0 × 10^−0^	
	Sex × age interaction	0.00101 (0.00067/0.00136)	0.146	8.5 × 10^−9^	
Serum phosphate (mmol/L)	Sex	−0.06262 (−0.10594/−0.01931)	−0.096	0.005	
	Age	−0.00363 (−0.004152/−0.00310)	−0.225	6.1 × 10^−41^	0.051
	Albumin	−0.00692 (−0.00802/−0.00582)	−0.152	1.4 × 10^−34^	
	Sex × age interaction	0.00201 (0.00123/0.00278)	0.178	3.8 × 10^−7^	
Calcium × phosphate (mmol^2^/L^2^)	Sex	−0.17317 (−0.27049/−0.07586)	−0.118	4.9 × 10^−4^	
	Age	−0.00882 (−0.01000/−0.00763)	−0.243	1.5 × 10^−47^	0.053
	Albumin	0.00688 (0.00441/0.00935)	0.067	4.9 × 10^−8^	
	Sex × age interaction	0.00568 (0.00394/0.00742)	0.225	1.7 × 10^−10^	
Corrected serum calcium (mmol/L)	Sex	−0.03428 (−0.05359/−0.01498)	−0.120	0.001	
	Age	−0.00022 (−0.00046/0.00001)	−0.032	0.06	0.010
	Sex × age interaction	0.00102 (0.00067/0.00136)	0.206	8.3 × 10^−9^	

## Discussion

In this study we found sex differences in serum calcium and phosphate that were age dependent with women above the age of 45 years having higher serum calcium and phosphate levels and a higher calcium × phosphate compared to men. Also, the levels in women above 45 years were higher compared to women < 45 years of age. No sexual dimorphism was found in the younger age groups. Sex differences in serum phosphate appeared on average 10 years before sex differences in serum calcium were observed. Furthermore, there was a significant interaction between age and sex for both serum calcium and phosphate in all three samples.

There are a few older and mostly small-sized studies that have found, although not always consistently, higher serum calcium levels in elderly women compared to men (13, 14, 15, 16). Several studies have consistently shown that postmenopausal women have higher serum phosphate levels than men of similar age (3, 13, 17, 18, 19, 20). Furthermore, several studies showed an age-related decline in serum phosphorus level with sex differences appearing around menopause, where in women serum phosphate increases around 40 years of age before decreasing again after the age of 60 (21, 22).

This study, as well as a population-based cohort study (W N H Koek *et al.*, unpublished observations) has shown that there is a clear age-related sex difference with respect to serum calcium and phosphate levels. These sex differences appear to evolve around the age of menopause, for serum phosphate and a decade later for serum calcium. Previously, it has been hypothesized that a fall in estrogens around menopause causes the appearance of sex differences in serum calcium and phosphate levels (23, 24). When a fall in estrogens is hypothesized to cause changes in serum calcium and phosphate levels one can speculate whether there is an opposite change during growth spurt when estrogen levels increase. Laboratories report different reference values during childhood for serum calcium and phosphate in boys and girls at different ages (emedicine.medscape.com; serum calcium and serum phosphate; reference values) which may indicate that there is a sex difference and potentially a relation between sex hormones and serum levels of calcium and phosphate. Several studies have shown serum phosphate levels to be highest in early infancy and to decrease during the course of childhood reaching adult values in late adolescence (25, 26, 27, 28). The higher phosphate levels in childhood are attributed to increased demands for adequate bone growth (29). In a study in 1984 Krabbe *et al*. have shown a relation of serum phosphate but not calcium levels with puberty development. Serum phosphate levels increased 12 to 9 months before peak height velocity and 3 months before the first pubic hair stage, Tanner stage 2 (30). In our study we did not find sex differences in serum calcium and phosphate in the youngest age group in any of the three samples. Due to the relative small number of subjects in the young groups we were not able to further stratify into smaller age group in order to assess if serum calcium and phosphate levels changed during different ages in childhood and whether this was sex dependent.

Since the largest differences in sex hormones between boys and girls exist during puberty, assessing the effect of the hormonal surge on serum calcium and phosphate levels will shed light on the relation between hormonal changes and serum calcium and phosphate levels.

Another interesting group of subjects to study the effects of hormones on calcium and phosphate homeostasis would be women during different times in their menstrual cycle to observe whether hormonal fluctuations influence serum calcium and phosphate levels.

This study was not set up to investigate the molecular pathways behind this age-dependent sexual dimorphism in serum calcium and phosphate levels.

### Relevance of findings and clinical implications

Our data do not only confirm reported age-related sex differences in serum phosphate but also strengthen earlier inconsistent finding of age-related sex differences in serum calcium levels. However, unlike previously mentioned studies, our study contains data from three different sample years of a hospital information system without further selection encompassing a wide age range from 1 to 99 years, therefore being a very close representation of a normal hospital and out-patient population. In this case the lack of selection emphasizes the robustness of the age- and sex-related differences. With sex differences more prominent on the research agenda in the last decade, there is a renewed interest in the relation of sex differences in health and disease. Despite the differences in our study being relatively small, the robustness of the data with similar findings in each of the three different cohorts, clearly points to age- and sex-related differences in physiology concerning calcium and phosphate homeostasis. The relevance and the clinical implications of observed sex differences, although they were relatively small, is that understanding the physiology of sex differences may aid in understanding age-related diseases with a clear sex difference that are associated with calcium and phosphate levels like cardiovascular diseases, metabolic syndrome, osteoporosis and also mortality (3, 13, 17, 18, 21, 31). The fact that women with osteoporosis are reported to have an increased risk for cardiovascular disease exemplifies this (32). Moreover, women with higher coronary artery calcification scores were found to have lower BMD, an association that is not found in men (33). Furthermore, a study using Mendelian randomization showed that a genetic predisposition for higher serum calcium was associated with increased coronary artery disease and myocardial infarction (34). These findings underscore the importance of sex differences of diseases like cardiovascular diseases and osteoporosis. Therefore, establishing sex differences in calcium and phosphate metabolism and understanding the physiological mechanisms that underlie these differences can aid in the further understanding of these diseases. Moreover, this could help with sex-specific strategies in prevention and treatment of common age-related diseases such as osteoporosis and cardiovascular diseases.

### Strengths and limitations

The strength of this study is the availability of data in a large number of men and women in the three samples evaluated. Inorganic phosphate and albumin assays have not changed over the years 2005 to 2015. However, for the calcium assay there has been a change in assay in 2013 to the GenII assay. Consequently, calcium levels showed a minimal deviation toward lower serum calcium values. This deviation is not expected to impact sex differences or age-related effects within the 2014 sample, but values obtained within 2014 cannot be compared to sample values obtained in the 2005 and 2010 sample group. Moreover, the measurement systems used to measure inorganic phosphate, albumin and total serum calcium, despite being from Roche, varied in these three sample years, therefore data of the 3 years were analyzed separately and comparing the years should be done with care. Nevertheless, result across these three sample years were very consistent.

Since we analyzed serum levels from a database of a hospital lab there may be a bias in the analyses because of underlying medical conditions that prompted the ordering of measurements of serum calcium and phosphate levels in these subjects. Since we had no information of these conditions, we were not able to take into account or exclude diseases that influence calcium and phosphate homeostasis. Therefore, one should be cautious with extrapolation of these results to a healthy population. Despite these flaws in the design of the study, the differences in older age subjects between the sexes were present at all three samples and are in line with our previous study where we assessed serum calcium and phosphate levels in an aging population (W N H Koek *et al.*, unpublished observations). Other limitations of our study are the lack of data on hormone levels such as estradiol and testosterone, and on other hormones that are more directly linked with calcium and phosphate metabolism including vitamin D, PTH and FGF23 levels that could also underlie the observed sex differences in serum calcium and phosphate levels.

## Conclusions

The results of this study showed that there is a consistent sex difference in serum calcium and phosphate levels and calcium × phosphate, and that this is age dependent with women ≥45 years of age having significantly higher serum levels of calcium, phosphate and calcium × phosphate compared to men of similar age and compared to women < 45 years of age. There are no sex differences in the age groups < 45 years. Furthermore, women but not men ≥45 years have higher serum calcium and phosphate levels compared to the group < 45 years thus suggesting that a decline in sex steroid levels after menopause is responsible for these sex differences. These findings may be of relevance for increased incidence of CVD in women after menopause.

## Supplementary Material

Supplemental figure: splines for serum phosphate and calcium levels in men and women, for each year sampled

## Declaration of interest

The authors declare that there is no conflict of interest that could be perceived as prejudicing the impartiality of the research reported.

## Funding

This work was supported by (NWO)-Research Institute for Diseases in the Elderly (Grant number 948-00-001).
