# Supplementary material for: Age-dependent sex differences in calcium and phosphate homeostasis
Source: Endocr Connect. 2021 Feb 4;10(3):273–82. doi: 10.1530/EC-20-0509 (PMC8052581; doi:10.1530/EC-20-0509)
Supplement: Supplemental figure: splines for serum phosphate and calcium levels in men and women, for each year sampled [file supplementary_figure_1.pdf]

Supplemental figure: splines for serum phosphate and calcium levels in men and women, for each year sampled

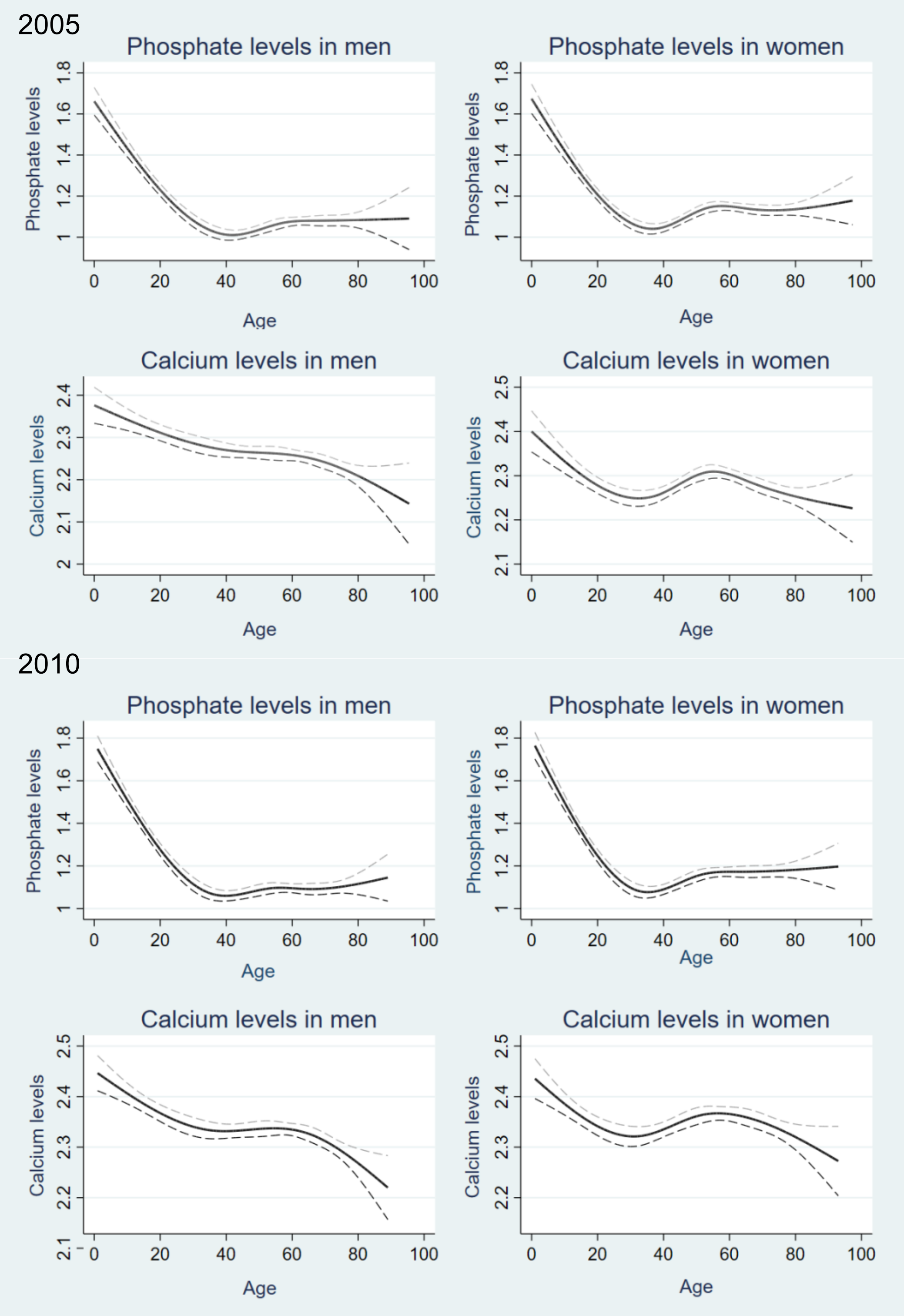

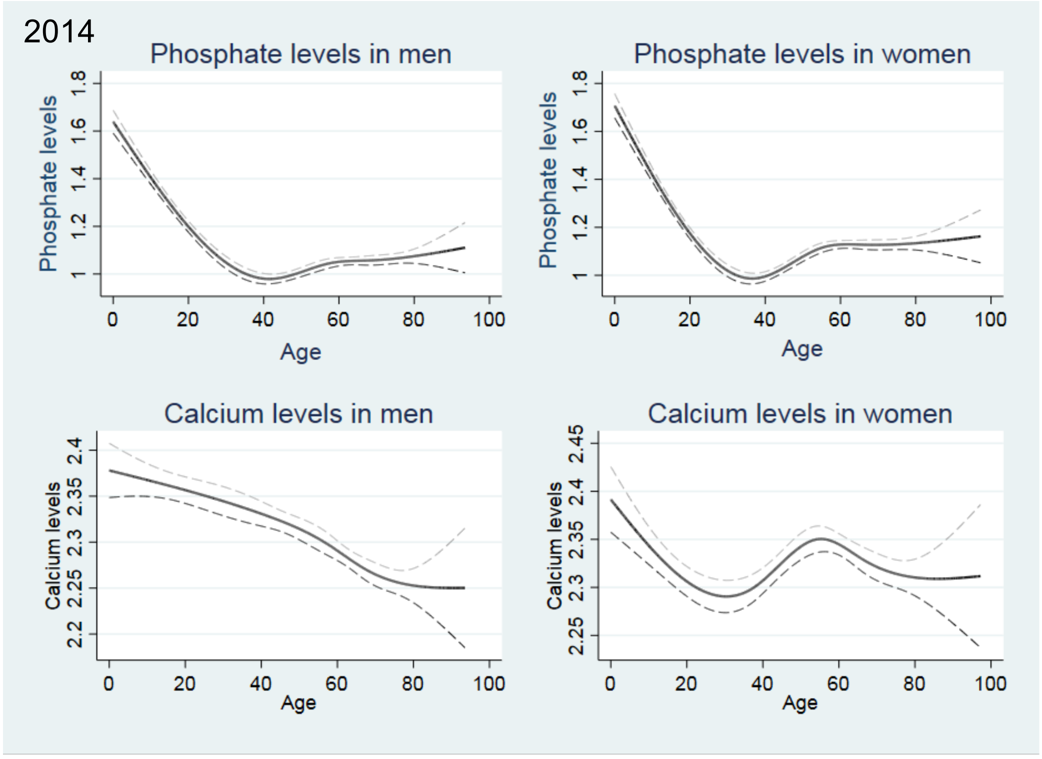
2014
Phosphate levels in men
Phosphate levels
1 1.2 1.4 1.6 1.8
0 20 40 60 80 100
Age
Phosphate levels in women
Phosphate levels
1 1.2 1.4 1.6 1.8
0 20 40 60 80 100
Age
Calcium levels in men
Calcium levels
2.2 2.25 2.3 2.35 2.4
0 20 40 60 80 100
Age
Calcium levels in women
Calcium levels
2.25 2.3 2.35 2.4 2.45
0 20 40 60 80 100
Age
